# Differences in the Composition of the Rumen Microbiota of Finishing Beef Cattle Divergently Ranked for Residual Methane Emissions

**DOI:** 10.3389/fmicb.2022.855565

**Published:** 2022-04-29

**Authors:** Paul E. Smith, Alan K. Kelly, David A. Kenny, Sinéad M. Waters

**Affiliations:** ^1^Teagasc, Animal and Bioscience Research Department, Animal and Grassland Research and Innovation Centre, Meath, Ireland; ^2^UCD School of Agricultural and Food Science, University College Dublin, Dublin, Ireland

**Keywords:** rumen, microbiota, 16S rRNA, cattle, methane

## Abstract

With the advent of high throughput technology, it is now feasible to study the complex relationship of the rumen microbiota with methanogenesis in large populations of ruminant livestock divergently ranked for enteric emissions. Recently, the residual methane emissions (RME) concept has been identified as the optimal phenotype for assessing the methanogenic potential of ruminant livestock due to the trait’s independence from animal productivity but strong correlation with daily methane emissions. However, there is currently a dearth of data available on the bacterial and archaeal microbial communities residing in the rumens of animals divergently ranked for RME. Therefore, the objective of this study was to investigate the relationship between the rumen microbiota and RME in a population of finishing beef cattle. Methane emissions were estimated from individual animals using the GreenFeed Emissions Monitoring system for 21 days over a mean feed intake measurement period of 91 days. Residual methane emissions were calculated for 282 crossbred finishing beef cattle, following which a ∼30% difference in all expressions of methane emissions was observed between high and low RME ranked animals. Rumen fluid samples were successfully obtained from 268 animals during the final week of the methane measurement period using a trans-oesophageal sampling device. Rumen microbial DNA was extracted and subjected to 16S rRNA amplicon sequencing. Animals ranked as low RME had the highest relative abundances (*P* < 0.05) of lactic-acid-producing bacteria (*Intestinibaculum*, *Sharpea*, and *Olsenella*) and *Selenomonas*, and the lowest (*P* < 0.05) proportions of *Pseudobutyrivibrio*, *Butyrivibrio*, and *Mogibacterium*. Within the rumen methanogen community, an increased abundance (*P* < 0.05) of the genus *Methanosphaera* and *Methanobrevibacter* RO clade was observed in low RME animals. The relative abundances of both *Intestinibaculum* and *Olsenella* were negatively correlated (*P* < 0.05) with RME and positively correlated with ruminal propionate. A similar relationship was observed for the abundance of *Methanosphaera* and the *Methanobrevibacter* RO clade. Findings from this study highlight the ruminal abundance of bacterial genera associated with the synthesis of propionate *via* the acrylate pathway, as well as the methanogens *Methanosphaera* and members of the *Methanobrevibacter* RO clade as potential microbial biomarkers of the methanogenic potential of beef cattle.

## Introduction

Recently, the Intergovernmental Panel on Climate Change (IPCC) has identified a rapid and sustained reduction in global methane production as a necessity to mitigate against the current increase in global temperatures (IPCC, 2021). At present, a third of the global anthropogenic methane emissions originate from ruminant livestock (Saunois et al., 2020). Indeed, enteric methane is accountable for ∼6% of global anthropogenic greenhouse gas (GHG) emissions (Gerber et al., 2013; Beauchemin et al., 2020) and ∼19% of Ireland’s national GHG emissions profile (Duffy et al., 2021). As a result, there has been an increased interest in the development of methane mitigation strategies for ruminant livestock.

To reduce the GHG emissions profile of the livestock industry, numerous authors have advocated the potential of genetic selection to achieve permanent and accumulative reductions to the methane output of future livestock generations (Wall et al., 2010; Pickering et al., 2015; de Haas et al., 2017; Beauchemin et al., 2020). To date, most investigations examining the relationship between the rumen microbiota and methane output have been conducted on animals ranked for daily methane emissions (DME; g/day; Danielsson et al., 2017) or methane yield (MY; g/kg of DMI; Kittelmann et al., 2014; Shi et al., 2014; Roehe et al., 2016). However, the direct genetic selection of animals for reduced DME or MY is unlikely to be implemented as part of a breeding strategy due to the antagonistic relationship of both traits with animal productivity (Bird-Gardiner et al., 2017; Renand et al., 2019; Smith et al., 2021).

Residual methane emissions (RME), defined as the difference between an animal’s actual and expected methane output based on its level of feed intake and body weight (Bird-Gardiner et al., 2017), has recently been advocated as the optimal trait for identifying low methane emitting cattle due to the trait’s phenotypic and genetic independence of animal productivity but strong correlation with DME (Herd et al., 2014; Manzanilla-Pech et al., 2016; Bird-Gardiner et al., 2017; Donoghue et al., 2020; Smith et al., 2021). Indeed, supported by moderate heritability estimates for the trait (Manzanilla-Pech et al., 2016; Donoghue et al., 2020), selecting animals for a low RME phenotype has the potential to reduce the methanogenic output of individual animals, without compromising the productivity of future generations of livestock.

Recently, our group observed a 30% difference in methane output along with shifts in theoretical hydrogen (H) production and a varied expression of microbial fermentation pathways associated with propionate production, yet a similar level of animal productivity, in cattle ranked low for RME (Smith et al., 2021). However, to date, there has been no investigation on the effect of ranking animals for RME on the composition of the rumen microbiota.

The abundance and fermentative activity of individual members of the rumen microbiota are influenced by fluctuating H dynamics in the rumen (Janssen, 2010), with methanogenesis recognised as one of the primary metabolic processes regulating dissolved ruminal dihydrogen (H_2_) concentrations (McAllister and Newbold, 2008; Morgavi et al., 2010). While the genetic factors controlling methanogenesis are yet to be determined, the composition of the rumen microbiota has explained 15–40% of the variation in methane output in some studies (Difford et al., 2018; Wallace et al., 2019). This suggests it may be possible to discover potential rumen microbial biomarkers that are reflective of the methanogenic potential of an animal. Indeed, the discovery of rumen microbial signatures associated with methanogenesis may benefit the identification of low-methane emitting animals by reducing the number of animals required to undergo long and expensive methane measurement estimation periods, as part of the development of an environmentally focused breeding programme.

Therefore, the objective of this study was to investigate the composition of the rumen microbiota in animals, phenotypically divergent in RME, in an effort to identify rumen microbial biomarkers indicative of the methanogenic potential of an animal.

## Materials and Methods

All animal procedures used in this study were approved by the Teagasc Animal Ethics Committee and conducted using procedures consistent with the experimental licence (AE19132/P078) issued by the Irish Health Products Regulatory Authority in accordance with European Union legislation (Directive 2010/63/EU) for the protection of animals used for scientific purposes.

### Animal Model

This experiment was conducted as part of a larger study designed to investigate the effects of ranking finishing beef cattle, in terms of RME, on enteric emissions and animal productivity. A detailed description of the animal model, measurements recorded, and derivation of traits has been presented in Smith et al. (2021).

Briefly, over a period of 18 months, data were obtained from 282 commercial beef cattle (steers = 128 and heifers = 154) enrolled in a feed efficiency performance test. Cattle were the progeny of AI bulls under evaluation as part of the Gene Ireland Breeding Program^1^, and were recruited from commercial breeding herds based on factors including sire, breed, genetic merit, pedigree, age, and performance tested under standardised conditions at the Irish Cattle Breeding Federation (ICBF) national beef bull progeny test station (Tully, Co., Kildare, Ireland). Cattle included in this study originated from continental late-maturing beef dams (Charolais, Limousin, or Simmental), sired by early maturing (EM) or late maturing (LM) sire breeds. The proportion of EM and LM sired animals was 25 and 75%, respectively.

Eligible cattle entered the test centre in groups of 40–75 animals, hereby referred to as “batches,” and underwent a minimum 100-day feed efficiency performance test. Starting in January 2019 and finishing in July 2020, animals from seven consecutive batches were included in this study. Upon arrival at the facility, cattle were allocated to indoor pens (6.1 m × 4.6 m) bedded with peat. Cattle were separated based on gender and initially penned in groups of five to six depending on their initial weight and age. Cattle were offered a 30-day adjustment period to allow dietary acclimatisation and adaption to the facilities. During the adjustment phase, animals were fitted with a RFID tag (HDX EID Tag, Allflex Livestock Intelligence, Dallas, TX, United States). Once tagged, the pen size was increased by opening the gates between adjacent pens to accommodate 11–30 animals per pen. The mean age and body weight of animals at the beginning of the measurement period were 441 days (SD = 49 days) and 476 kg (SD = 67 kg), respectively. Steers and heifers averaged 476 (SD = 46 days) and 410 (SD = 27 days) days of age while LM and EM averaged 442 (SD = 51 days) and 435 (SD = 43 days) days of age at the commencement of the measurement period, respectively.

Cattle were offered the same total mixed ration (TMR) *ad libitum*, which consisted of 77% concentrates and 23% hay (see Supplementary Table 1), and underwent a mean feed intake measurement period of 91 days (71–128 days). Enteric emissions (methane and carbon dioxide) were estimated over a 21-day period during a mean feed efficiency test period of 91 days using the GreenFeed Emissions Monitoring system (GEM; C-Lock Inc., Rapid City, SD, United States). Following the completion of the measurement period, animals were slaughtered in a commercial abattoir.

### Rumen Fluid Collection

During the last week of the enteric emissions measurement period, samples of rumen fluid (25–50 ml) were collected from 268 animals before feeding using the transoesophageal rumen sampling device (FLORA rumen scoop; Guelph, ON, Canada). Feed was restricted from animals for a minimum of 2 h before sampling. Samples were divided across two 25-ml tubes with ruminal fluid pH measured immediately using a digital pH meter (Orion SA 720; Thermo Fisher Scientific, Waltham, MA, United States). Following this, 500 μl of rumen fluid was pipetted into 2-ml cyrotubes (Sarstedt, Co., Wexford, Ireland) containing autoclaved zirconia beads (0.3 g of 0.1 mm and 0.1 g of 0.5 mm) and immediately preserved *via* snap-freezing in liquid nitrogen along with the remaining rumen fluid contained in 25 ml tubes. On the same day of sampling, samples were transported 61 km to the Teagasc research facility (Teagasc Grange, Dunsany, Co., Meath, Ireland) on dry ice and stored at –80°C until further molecular analysis was conducted.

### DNA Extraction and Library Preparation

Eight samples were misplaced resulting in microbial DNA being extracted from 260 samples of 500 μl of frozen rumen fluid sample using a modified version of repeated bead beating and column purification method (Yu and Morrison, 2004), as previously described (McGovern et al., 2018; Smith et al., 2020a,b). A blank extraction control was subjected to the same procedure as rumen fluid samples for each extraction kit. DNA quality was assessed using agarose gels (0.8%) and a 1-kb DNA ladder (Bioline GmbH, Luckenwalde, Germany). The concentration of extracted DNA was quantified on the Nanodrop 1000 spectrophotometer and diluted to 100 and 5 ng/μl before running agarose gels and PCR amplification.

Using 12.5 ng of extracted rumen microbial DNA, amplicon libraries (*n* = 260) were generated by performing two rounds of PCR amplification as outlined in the Illumina Miseq *16S Sample Preparation Guide* with minor modifications to cycle length, as outlined in McGovern et al. (2018) and Smith et al. (2020c). In addition, six amplicon libraries were generated to assess sequencing run performance and library preparation. Three amplicon libraries were generated using the ZymoBIOMICS*™* Microbial Community DS (Zymo Research Corp., Irvine, CA, United States). An additional three libraries were synthesised using the synthetic rumen amplicon sequencing standard, as described by Smith et al. (2020c).

The first round of PCR amplification, targeting the V4 hypervariable region of the 16S rRNA gene, was performed using the 515F/806R primers (Caporaso et al., 2011) designed with Nextera overhang adapters and 2x KAPA Hifi HotStart ReadyMix DNA polymerase (Roche Diagnostics, West Sussex, United Kingdom). Cycle conditions were as follows: 95°C for 3 min, 20 cycles at 95°C for 30 s, 55°C for 30 s, 72°C for 30 s, and then 72°C for 5 min.

Amplicons were purified using the QIAquick PCR Purification Kit (Qiagen, Manchester, United Kingdom). A negative control subjected to the same procedures as rumen amplicon samples was performed for each purification kit. Following purification, amplicons were subject to a second round of PCR to permit the attachment of dual indices and Illumina sequencing adapters using the Nextera XT indexing kit (Illumina, San Diego, CA, United States). Cycle conditions for the second round of PCR were 95°C for 3 min, 8 cycles at 95°C for 30 s, 55°C for 30 s, 72°C for 30 s and then 72°C for 5 min followed by an additional PCR purification with the QIAquick PCR Purification Kit (Qiagen, Manchester, United Kingdom). Confirmation of amplicon generation was conducted visually on a 2% agarose gel. Amplicons were split across three separate runs, pooled together in equal concentration, and subject to gel purification using the Qiagen Gel Extraction Kit (Qiagen, Manchester, United Kingdom) to remove adapter primers followed by further purification to remove any residues of agarose using the QIAquick PCR purification kit (Qiagen, Manchester, United Kingdom). In total, 276 samples were sequenced (*n* = 260 rumen samples; *n* = 6 positive controls; *n* = 11 negative controls).

Pooled sample purity and quantity were analysed on the Nanodrop 1000 with further validation on the Qubit fluorometer and using the KAPA SYBR FAST universal kit with Illumina Primer Premix (Roche Diagnostics, West Sussex, United Kingdom). Following this, the library pool was diluted and denatured as per the Illumina Miseq *16S Sample Preparation Guide* with sequencing conducted on the Illumina MiSeq using the 500 cycle version 2 MiSeq reagent kit (Illumina, San Diego, CA, United States) over three separate runs.

### Rumen Metabolite Analysis

Short-chain fatty acid concentrations in rumen fluid samples were measured using a Varian Saturn 2000 GC 450 (Varian, Middelburg, The Netherlands). A detailed description of sample preparation, the extraction of VFA, and the cycle conditions utilized were previously described (Smith et al., 2021).

### Sequencing Analysis

Amplicon sequence data were processed in R (version 4.0.2) using DADA2 (version 1.18.0) and submitted to the pipeline as described by Callahan et al. (2016) with minor modifications. Quality checks of both forward and reverse reads were initiated followed by the filtering and trimming of poor quality reads and removal of primer sequences using the trimLeft function. Identical sequences were combined using the dereplication function followed by the merging of forward and reverse reads. An ASV table was then constructed following which chimeric sequences were removed and taxonomy assigned to sequences variants using the RefSeq + RDP (NCBI RefSeq 16S rRNA database supplemented by RDP; release date 06/11/2020) downloaded from the DADA2 website. A bootstrapping threshold of 80 was applied for taxonomic classification by incorporating minBoot = 80 as part of the assignTaxonomy function. Sample metadata, sequence taxonomy, and ASVs were combined into a phyloseq object using phyloseq (version 1.34.0; McMurdie and Holmes, 2013) for further analysis. Ten rumen amplicon samples were removed because they had a significantly low sequencing depth. A rarefication curve was plotted for the remaining rumen samples (*n* = 250). Based on a plateauing of the generated rarefication curve (see Supplementary Figure 1), samples were rarefied to the lowest sequencing depth of all samples (26,366 reads per sample). Following this, alpha (Shannon and Simpson) diversity was calculated for each sample. For comparisons of beta diversity, as well as differential abundance analysis, ASVs which were not present in >5% of the samples were removed before calculating the relative abundance, based on rarefied reads.

To determine the proportion of rumen methanogens belonging to the SGMT or RO clade, ASVs assigned to the *Methanobrevibacter* genus were further classified by conducting an online NCBI BLAST search against the RefSeq database^2^.

### Data and Statistical Analysis

Before assessing differences in the bacterial and archaeal structure amongst RME groups, the homogeneity of group dispersions was assessed between low and high-ranked animals. Following this, PERMANOVA tests based on Bray-Curtis dissimilarities, 9,999 permutations, and a significance level of (*P* < 0.05) were implemented to determine if the bacterial and archaeal structure differed amongst high and low RME animals. Both the assessment of the homogeneity of group dispersions and PERMANOVA analysis were carried using the R package vegan (Oksanen et al., 2019) (version 2.5.7) implemented through microbiome (Lahti and Shetty, 2017) (version 1.12.0). The R package plotly (version 4.9.3) was used to generate 3D NMDS plots based on Bray-Curtis dissimilarities.

Statistical comparisons of the relative abundance of the rumen bacteria and archaea between the RME groups was conducted on ASVs with a mean relative abundance greater than 0.5% in at least one RME group using the GLIMMIX procedure of SAS (SAS Inst. Inc., Cary, NC; version 9.4). The statistical model used included the fixed effect of RME group (high, medium, and low), breed maturity/genotype (LM and EM), gender (steer and heifer), and their interactions. Non-statistically significant (*P* > 0.10) interactions were subsequently excluded from the final model. Age and initial body weight at the start of the performance test were included as covariates, and a contemporary group was incorporated as a random effect in the statistical model. The residuals of each model were normally distributed. Differences among means were determined by *F*-tests using Type III sums of squares. The PDIFF option and the Tukey test were applied to evaluate pairwise comparisons between means. Mean values were considered to be different when *P* < 0.05 and a tendency when *P* ≥ 0.05 and <0.10. The associations among the microbial abundances with performance and fermentation parameters were determined through partial correlations, adjusted for gender, breed maturity, and contemporary group using the MANOVA/PRINTE statement within the GLM procedure of SAS. Correlation coefficients were classified as strong (*r* > 0.6), moderate (*r* between 0.4 and 0.6), or weak (*r* < 0.4), respectively.

## Results

### Animal Performance

Statistical comparisons of feed intake and emissions traits amongst the RME groups are presented in Supplementary Table 2. Low RME animals produced 17.69 and 30.42% less (*P* < 0.05) DME in comparison to animals ranked as medium and high for RME, respectively. Similarly, the low RME animals had lower (*P* < 0.05) daily carbon dioxide emissions (DCE; kg/day) than animals ranked as medium and high. Low RME animals had the lowest (*P* < 0.05) MY and methane intensity (MI; g/kg of carcass weight) of the RME groups. A difference of 29.73 and 29.63% for MY and MI was detected amongst the low and high RME groups, respectively.

### DNA Extraction and Sequencing Performance

After quality filtering, merging, and removal of chimeric sequences, an average of 67,970 ± 27,857 reads per rumen sample were generated across the three runs. Correlations between the composition of libraries generated from the DS standards and the theoretical composition of the ZymoBIOMICS™ ranged from *r*_*s*_ 0.95 to 0.97. In addition, the correlation of the synthetic sequencing standard with the composition reported in Smith et al. (2020c) ranged from *r*_*s*_ 0.89 to 0.90. Negative extraction controls generated on average 50 reads per sample (range of 13–93) after quality filtering, merging, and removal of chimeric sequences. DNA extraction and sequencing performance were deemed satisfactory based on the strong concordance of the microbial compositions in both standards with that of their theoretical composition and the low number of reads obtained for negative controls.

### Rumen Microbial Composition

**FIGURE 1:**
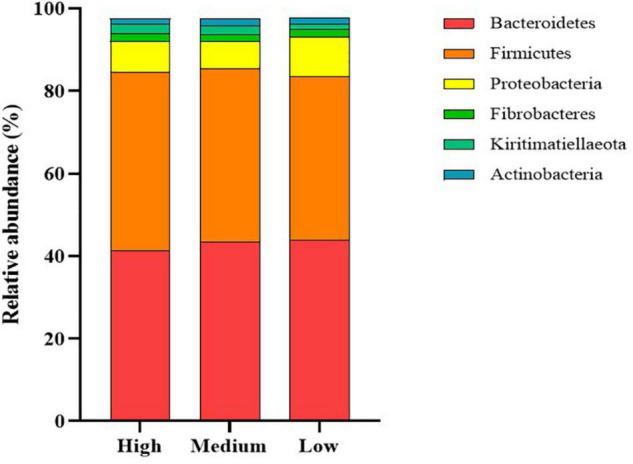
Stack plot comparing differences, in the relative abundances of the six most abundant rumen bacterial phyla, between cattle ranked as high, medium, and low for residual methane emissions.

**FIGURE 2:**
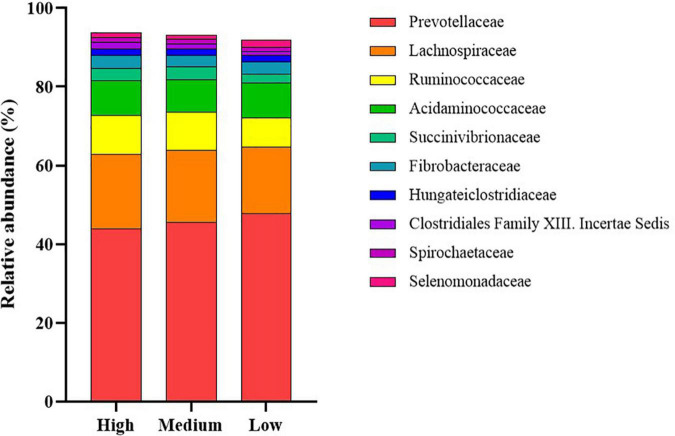
Stack plot comparing differences, in the relative abundances of the ten most abundant rumen bacterial families, between cattle ranked as high, medium, and low for residual methane emissions.

The dominant members of the bacterial community, within each group at the phylum and family levels, are displayed in Figures 1, 2. At the phylum level, the rumen bacterial community was dominated by *Firmicutes* and *Bacteroidetes*, which had a mean combined relative abundance of 84.58%. *Proteobacteria* was the next most abundant bacterial phylum (7.70%) followed by *Fibrobacteres* (1.89%), *Kiritimatiellaeota* (1.86%), and *Actinobacteria* (1.53%). *Prevotellaceae* was the most abundant bacterial family observed, with a mean relative abundance of 45.73% across all samples. The families *Lachnospiraceae, Ruminococcaceae*, and *Acidaminococcaceae* contributed to 35.72% of the bacterial community composition. *Prevotella* was the primary genus of bacteria observed with a mean relative abundance of 55.05% across all samples, followed by *Succiniclasticum* (11.33%), *Ruminococcus* (9.28%), *Fibrobacter* (4.15%), and *Succinivibrio* (2.27%).

The genera *Methanobrevibacter* and *Methanosphaera* accounted for 93.87 and 5.09%, respectively, of the archaeal community across all samples. Within the *Methanobrevibacter* genus, the relative abundance of members of SGMT and RO clade was 51.31 and 46.58%, respectively, with the remaining 2.11% of species identified as *M. boviskoreani*.

### Effect of Residual Methane Emissions Ranking on Bacterial Community Composition

**FIGURE 3:**
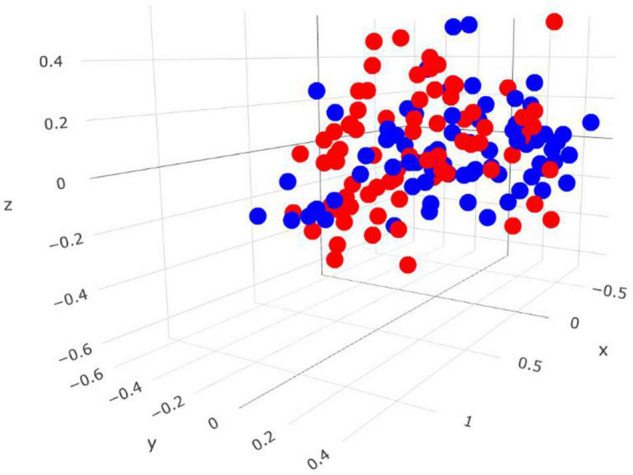
Three-dimensional Bray-Curtis NMDS plot highlighting differences in the bacterial community composition between animals ranked as high and low for residual methane emissions. Red = low RME; blue = high RME.

Comparisons in the bacterial community, at the genus level, between RME groups, are presented in Table 1. The overall bacterial community structure did not differ between high and low-ranked RME animals (PERMANOVA; *P* = 0.87) (Figure 3). In addition, no difference in *alpha* diversity was detected at the species level between the RME groups (*P* > 0.05). At the phylum level, an increased (*P* < 0.05) relative abundance of *Proteobacteria* was observed in the low compared to high RME animals. The opposite was observed for the abundance of *Kiritimatiellaeota*, with an increased (*P* < 0.05) abundance observed in the high RME group. No other bacterial phyla were impacted by RME ranking.

**TABLE 1:** Characterization of the rumen bacterial genera in finishing beef cattle ranked for residual methane.

	RME Ranking^1^				
Bacteria genus	High	Medium	Low	SEM^2^	*P*-value
	*n* = *80*	*n* = *96*	*n* = *74*		
*Anaeroplasma*	0.71	0.77	0.76	0.11	0.59
*Bifidobacterium*	1.63	1.81	1.72	0.3	0.77
*Butyrivibrio*	1.35^a^	1.12^a^	0.80^b^	0.19	<0.001
*Eubacterium*	0.55	0.56	0.68	0.07	0.06
*Fibrobacter*	4.35	4.21	4.08	0.5	0.77
*Intestinibaculum*	0.37^a^	0.50^a^	0.86^b^	0.1	<0.0001
*Mogibacterium*	1.63^a^	1.16^b^	0.97^b^	0.16	<0.0001
*Olsenella*	0.53^a^	0.74^a^	1.02^b^	0.13	<0.0001
*Prevotella*	53.15	54.55	55.71	1.74	0.23
*Pseudobutyrivibrio*	0.69^a^	0.52^b^	0.43^b^	0.09	<0.001
*Ruminobacter*	1.77	1.61	1.08	0.43	0.08
*Ruminococcus*	9.92	9.3	8.65	0.67	0.16
*Selenomonas*	0.74^a^	0.85^a^	1.21^b^	0.15	<0.001
*Sharpea*	1.27^a^	1.75^ab^	1.86^b^	0.26	0.03
*Succiniclasticum*	11.48	11.26	10.51	0.69	0.23
*Succinivibrio*	2.78	2.41	1.84	0.46	0.41
*Treponema*	1.63	1.61	1.56	0.17	0.82

*^1^ High = RME was > 0.5 SD above the mean; Medium = RME was ± 0.5 SD above and below the mean; Low = RME was > -0.5 SD below the mean.*

*^2^ SEM = pooled standard error.*

*^a,b^Least squares means within main effect and a row with different superscripts differ.*

At the family level, the proportions of *Ruminococcaceae*, *Succinivibrionaceae*, and *Clostridiales Family XIII Incertae Sedis* were increased (*P* < 0.05) in the high compared to low RME ranked animals. The proportions of the bacterial families, *Lactobacillaceae*, *Erysipelotrichaceae*, and *Selenomonadaceae* were greater (*P* < 0.05) in low RME groups in comparison to their high counterparts.

Within the bacterial family *Lachnospiraceae*, greater (*P* < 0.05) proportions of both *Pseudobutyrivibrio* and *Butyrivibrio* were present in high RME ranked animals. Animal ranking for RME had a significant influence on the genus *Mogibacterium* (*P* < 0.05), which was significantly increased in high RME ranked animals. The proportion of the genus *Ruminobacter* tended (*P* = 0.08) to be higher in high RME animals. The bacterial genera *Intestinibaculum*, *Olsenella*, *Selenomonas*, and *Sharpea* had increased (*P* < 0.05) relative abundances in low RME ranked animals. In addition, the abundance of *Eubacterium* tended (*P* = 0.06) to be affected by RME ranking, with a decreased proportion of the genus observed in the high and medium ranked animals relative to low RME counterparts.

### Relationship of Rumen Bacteria With Enteric Emissions and Rumen Fermentation

Partial correlation analysis amongst the relative abundance of rumen bacterial genera with both enteric emissions traits and VFAs are presented in Tables 2, 3. The relative abundances of *Intestinibaculum*, *Olsenella*, and *Selenomonas* were negatively correlated (*P* < 0.05) with all methane phenotypes and positively correlated with propionate percentage. In addition, both *Intestinibaculum* and *Olsenella* were negatively associated (*P* < 0.05) with A:P ratio, along with *Olsenella* sharing a negative relationship (*P* < 0.05) with butyrate percentage. *Prevotella* abundance was negatively correlated (*P* < 0.05) with DME, RME, and MI. The abundances of *Butyrivibrio*, *Pseudobutyrivibrio*, *Mogibacterium*, and *Succiniclasticum* were positively correlated (*P* < 0.05) with all methane emission traits. Furthermore, the relative abundances of both *Butyrivibrio* and *Mogibacterium* were negatively associated (*P* < 0.05) with propionate percentage. The abundances of both *Ruminobacter* and *Ruminococcus* were positively correlated (*P* < 0.05) with both DME and RME. Both the proportions of *Fibrobacter* and *Treponema* were negatively associated with total SCFA production.

**TABLE 2:** Correlation coefficients amongst rumen bacterial genera and traits associated with enteric emissions.

	DME	DCE	RME	MY	*MI*
*Anaeroplasma*	–0.02	–0.04	–0.04	–0.07	0.01
*Bifidobacterium*	0.05	–0.01	0.03	0.00	0.10
*Butyrivibrio*	0.35***	0.10	0.30***	0.24**	0.28***
*Eubacterium*	–0.08	0.03	−0.13*	–0.10	–0.01
*Fibrobacter*	0.06	0.02	0.07	0.03	0.01
*Intestinibaculum*	−0.30***	0.01	−0.36***	−0.38***	−0.27***
*Mogibacterium*	0.38***	0.07	0.38***	0.38***	0.35***
*Olsenella*	−0.30***	0.00	−0.32***	−0.29***	−0.31***
*Prevotella*	−0.19**	–0.09	−0.13*	–0.09	−0.15*
*Pseudobutyrivibrio*	0.32***	0.12†	0.27***	0.22**	0.28***
*Ruminobacter*	0.13*	0.01	0.16*	0.15*	0.12†
*Ruminococcus*	0.23**	0.12†	0.14*	0.09	0.23**
*Selenomonas*	−0.25***	–0.02	−0.23**	−0.18**	−0.24**
*Sharpea*	–0.05	–0.01	–0.09	–0.10	–0.06
*Succiniclasticum*	0.13*	0.03	0.14*	0.15*	0.13*
*Succinivibrio*	0.12†	0.01	0.13*	0.09	0.11†
*Treponema*	0.11†	0.10	0.04	–0.01	0.03

*DME, daily methane production; DCE, daily carbon dioxide production; RME, residual methane emissions; MY, methane yield; MI, methane intensity.*

*†P < 0.10.*

* P < 0.05.

** P < 0.01.

*** P < 0.001.

**TABLE 3:** Correlation coefficients amongst rumen bacterial genera with rumen fermentation characteristics.

	Total SCFA (mM)	Acetate (%)	Butyrate (%)	Propionate (%)	A:P
*Bifidobacterium*	0.21**	−0.14*	0.18**	–0.03	–0.02
*Butyrivibrio*	–0.08	0.05	0.01	−0.16*	0.11
*Eubacterium*	0.06	–0.05	–0.04	0.04	–0.09
*Fibrobacter*	−0.24**	0.05	0.00	–0.03	0.02
*Intestinibaculum*	–0.08	−0.15*	–0.11	0.21**	−0.25**
*Mogibacterium*	–0.07	0.00	0.13†	−0.15*	0.11
*Olsenella*	–0.08	–0.04	−0.22**	0.16*	−0.16*
*Prevotella*	0.04	0.02	–0.04	0.10	–0.01
*Pseudobutyrivibrio*	−0.12†	0.04	0.02	−0.14†	0.09
*Ruminobacter*	–0.04	0.02	–0.05	0.02	–0.01
*Ruminococcus*	0.10	–0.09	0.05	–0.04	–0.02
*Selenomonas*	0.04	–0.06	0.03	0.14*	–0.09
*Sharpea*	0.03	–0.10	–0.04	0.03	–0.13
*Succiniclasticum*	0.03	0.09	0.09	–0.15	0.10
*Succinivibrio*	0.04	0.05	0.02	–0.08	0.05
*Treponema*	−0.18**	0.06	0.00	–0.09	0.06

*A:P = acetate to propionate ratio.*

*†P < 0.10.*

* P < 0.05.

** P < 0.01.

### Effect of Residual Methane Emissions Ranking on Archaeal Community Composition

**FIGURE 4:**
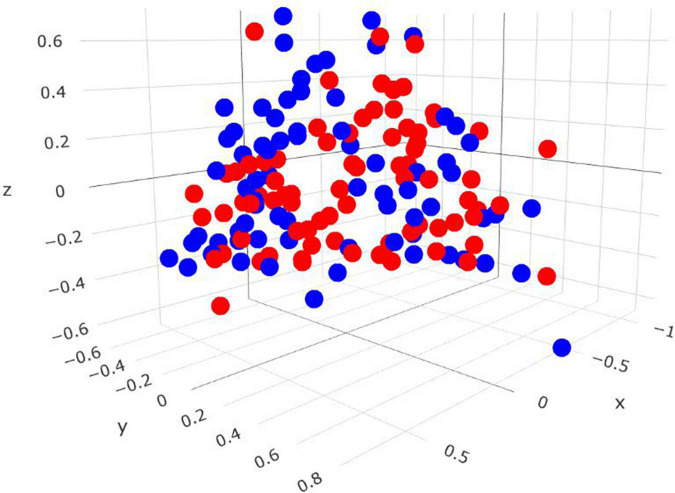
Three-dimensional Bray-Curtis NMDS plot highlighting differences in the archaeal community composition between animals ranked as high and low for residual methane emissions. Red = low RME; blue = high RME.

Comparisons in the archaeal community at the genus level and within the *Methanobrevibacter* clades between RME groups are presented in Table 4. Based on PERMANOVA analysis, a tentative difference in the structure of the archaeal community was detected amongst the high and low RME groups (*P* = 0.07), although no clear separation was observed (Figure 4). The relative abundance of *Methanobrevibacter* did not differ amongst the RME groups (*P* > 0.05). Within *Methanobrevibacter*, the abundance of the SGMT clade was not affected by RME ranking. However, an increased (*P* < 0.05) relative abundance of the RO clade was observed in the low compared to high RME animals. The relative abundance of *Methanosphaera* was increased (*P* < 0.05) in the low relative to high RME animals.

**TABLE 4:** Characterization of the rumen methanogens in finishing beef cattle ranked for residual methane.

	RME ranking^1^				
Rumen methanogens	High	Medium	Low	SEM^2^	*P*-value
	*n* = *80*	*n* = *96*	*n* = *74*		
**Genus**					
*Methanobrevibacter*	94.01	93.64	93.6	0.32	0.27
*Methanosphaera*	4.93^a^	5.22^a^	5.79^b^	0.39	<0.01
**Methanobrevibacter clades**					
RO	45.25^a^	48.07^ab^	53.92^b^	4.57	<0.01
SGMT	52.11	49.6	45.59	4.45	0.08

*^1^ High = RME was > 0.5 SD above the mean; Medium = RME was ± 0.5 SD above and below the mean; Low = RME was > -0.5 SD below the mean.*

*^2^ SEM = pooled standard error.*

*^a,b^ Least squares means within main effect and a row with different superscripts differ.*

### Relationship of Rumen Methanogens With Enteric Emissions and Rumen Fermentation

Partial correlation analysis amongst the relative abundance of rumen methanogens and with both enteric emissions and VFAs are presented in Tables 5, 6. The RO clade was negatively correlated (*P* < 0.05) with all methane traits, butyrate percentage, and A:P ratio, but was positively associated with propionate percentage. The opposite relationships were observed for the relative abundance of the SGMT clade, which was positively correlated (*P* < 0.05) with RME, MY, butyrate percentage, and A:P ratio, but negatively correlated with propionate percentage. *Methanosphaera* was negatively correlated (*P* < 0.05) with all methane phenotypes, acetate percentage, and the A:P ratio but positively correlated with propionate percentage.

**TABLE 5:** Correlation coefficients amongst rumen methanogens and traits associated with enteric emissions.

	DME	DCE	RME	MY	MI
**Genus**					
*Methanobrevibacter*	0.06	0.00	0.10	0.12†	0.10
*Methanosphaera*	−0.20**	–0.03	−0.23**	−0.22**	−0.19**
**Methanobrevibacter clades**					
RO	−0.14*	0.12†	−0.26***	−0.28***	−0.13*
SGMT	0.08	−0.15*	0.20**	0.23**	0.08

*DME, daily methane production; DCE, daily carbon dioxide production; RME, residual methane emissions; MY, methane yield; MI, methane intensity.*

*†P < 0.10.*

* P < 0.05.

** P < 0.01.

*** P < 0.001.

**TABLE 6:** Correlation coefficients amongst rumen methanogens with rumen fermentation characteristics.

	Total SCFA (mM)	Acetate (%)	Butyrate (%)	Propionate (%)	A:P
**Genus**					
*Methanobrevibacter*	0.12†	0.07	0	–0.06	0.12†
*Methanosphaera*	–0.05	−0.14*	0.01	0.14*	−0.22**
**Methanobrevibacter clades**					
RO	0.03	–0.1	−0.29***	0.18*	−0.21**
SGMT	0.01	0.08	0.26**	−0.15*	0.17*

*A:P = acetate to propionate ratio.*

*†P < 0.10.*

* P < 0.05.

** P < 0.01.

*** P < 0.001.

A strong negative correlation (*r* = –0.80; *P* < 0.0001) between the relative abundance of *Methanobrevibacter* and *Methanosphaera* was observed. A similar relationship was detected between the relative abundance of the SGMT and RO clade (*r* = –0.93; *P* < 0.0001).

## Discussion

Recently, RME has been identified as the optimal phenotype for identifying low methane emitting ruminants, with our group having recently reported an ∼30% difference in methane output, but comparable levels of animal productivity between high and low RME ranked animals (Smith et al., 2021). Indeed, the independence of RME from animal productivity makes it the ideal phenotype for examining the inherent biological mechanisms influencing methanogenesis. However, before this study, there had been no attempts to investigate the key rumen microbes associated with inter animal divergence in RME.

Animals ranked as low for RME had increased abundances of the lactic-acid-producing bacteria *Intestinibaculum*, *Olsenella*, and *Sharpea.* In addition, the abundances of *Intestinibaculum* and *Olsenella* were negatively associated with all methane phenotypes, but correlated positively with ruminal propionate percentage. This suggests that an increased abundance of both *Intestinibaculum* and *Olsenella* may play a role in reducing the dissolved H_2_ concentrations in the rumen *via* the redirection of metabolic H toward the acrylate pathway. Although *Intestinibaculum* and *Olsenella* are predominantly lactic-acid-producing bacteria (Kim et al., 2019) and not deemed to be among the potent producers of propionate in the rumen, members of *Selenomonas* are capable of fermenting lactate to propionate (Evans and Martin, 1997; Denman et al., 2015). This possibly explains the observed relationship of *Selenomonas* with propionate synthesis and methane output. Although this symbiotic relationship between lactic-acid-producing bacteria and *Selenomonas* will need further interpretation, it gives credence to the concept of “microbial teams” having a role in redirecting metabolic H to lactate and propionate, as proposed by Ungerfeld (2020).

Metabolic H can also be redirected toward butyrate production when H_2_ concentrations are high in the rumen; however, this will still result in a net production of H (Janssen, 2010). Elevated relative abundances of the genera *Pseudobutyrivibrio* and *Butyrivibrio*, known producers of formate, butyrate, acetate, and H_2_ (Van Gylswyk et al., 1996, Kelly et al., 2010; Emerson and Weimer, 2017; Palevich et al., 2017) in the rumen, were observed in high RME animals. Data generated *in vitro* suggests the existence of a symbiotic relationship between the species of *Butyrivibrio* and *Methanobrevibacter* associated with the utilisation of formate during hydrogenotrophic methanogenesis (Leahy et al., 2010). *Methanobrevibacter* species are also postulated to be capable of adhering to the surface of some *Butyrivibrio* species, which may benefit H_2_ transfer from bacteria to archaea, akin to the symbiotic relationship observed between ruminal protozoa and methanogens (Ng et al., 2016). As such, the catabolic activities of some members of the *Butyrivibrio* genus may indirectly support methanogenesis through the supply of formate to ruminal methanogens.

The positive relationship of *Mogibacterium* with RME follows a similar relationship to that of previous findings, showing an increased abundance of *Mogibacterium* to be associated with high MY cattle (Wallace et al., 2015). As this genus has been identified as being asaccharolytic (Nakazawa et al., 2000), its contribution to ruminal methanogenesis will require further investigation.

Traditionally, the expression of the methyl coenzyme M reductase (*mcr*) gene in the rumen has been advocated as a more credible biomarker of ruminal methanogenesis in comparison to metataxonomic-based applications (Wilkins et al., 2015). Moreover, recent evidence is supportive of the abundance of individual members of the rumen methanogen community as indicators for the methanogenic potential of an animal (Tapio et al., 2017; Ramayo-Caldas et al., 2020). Similar to the findings in this study, an elevated proportion of the *Methanobrevibacter* RO clade and *Methanosphaera* has been associated with a reduced methane output in sheep (Shi et al., 2014; Kittelmann et al., 2014) and dairy cows (Danielsson et al., 2017). Martínez-Álvaro et al. (2020) suggested an increased diversity of the methanogen community and methanogenesis pathway expression to contribute to a reduced MY in cattle. In this study, the structure of the methanogen community tended to differ amongst RME groups, with the abundance of *Methanosphaera* and the *Methanobrevibacter* RO clade increased in low RME animals. Members of the *Methanosphaera* genus predominately produce methane *via* the reduction of methanol (Tapio et al., 2017). In addition, the division of the two clades of *Methanobrevibacter* was proposed based on the ability of the SGMT clade to synthesise two isomers of the *mcr* gene, *mcrI* and *mcrII*, with only *mcrI* expressed by the RO clade (Tapio et al., 2017). A fluctuation in the abundance of methanogens, with differing methanogenesis pathways, between high and low methane emitting animals is often perceived as competition for methanogenesis substrates amongst ruminal methanogens for H_2_ (Morgavi et al., 2010). Consistent with this, a negative correlation between the relative abundances of SGMT and RO, as well as opposing correlations with RME was observed in this study, which may suggest competition amongst these members of the *Methanobrevibacter* genus for H_2_.

Furthermore, the reduction of methanol *via* methylotrophic methanogenesis has a lower H_2_ requirement in comparison to the hydrogenotrophic synthesis of methane, with the energetic advantage methylotrophic methanogens possess at low H_2_ pressure detailed by Feldewert et al. (2020). The availability of dissolved H_2_ in the rumen is also postulated to regulate the expression of the *mcr*, with *mcrII* only expressed when the quantity of H_2_ is high (Reeve et al., 1997) which, as depicted by Danielsson et al. (2017), gives the SGMT clade a competitive advantage in the presence of a greater availability of H_2_. Therefore, as more H_2_ is diverted to additional H sinks under high H_2_ concentrations, there will be a reduced production of H_2_ from fermentation, which, in the current experiment, may have increased the competitiveness of *Methanosphaera* in the rumen of low RME animals. Similarly, when dissolved H_2_ concentrations are kept low by an increased rate of methanogenesis and H_2_ producing fermentation pathways are favoured, the SGMT clade may have a competitive advantage over members of the RO clade as more H_2_ is produced during fermentation. Subsequently, the relationship between *Methanosphaera* and the abundances of the SGMT and RO clade with DME and RME observed in this study would suggest that the methanogenic output of an animal, at least in part, influences the composition of the rumen methanogen community, likely as a result of H dynamics in the rumen.

Recently, the abundances of some members of the rumen methanogen community associated with RME in this study have been proposed to be influenced by the genetics of the host. For example, the ratio of *Mbb. gottschalkii* to *Mbb. ruminantium* in the rumen has an estimated heritability of 0.17 (Li et al., 2019), while the abundance of *Methanosphaera* may also be regulated by host genetics (Difford et al., 2018). Based on the prospects of host genetics regulating the abundance of the methanogen community, it is likely the host may elicit some control over the rate of ruminal methanogenesis and/or concentration of dissolved H_2_. However, further investigation will be required to uncover the means by which the host regulates control over the prevailing conditions within the rumen.

Under the intensive finishing conditions deployed in this experiment, a baseline assessment of the relationship of the rumen microbiota with RME is presented. Furthermore, this study has identified some of the key microbial genera to target as part of the development of environmentally focused breeding programmes and future antimethanogenic dietary supplements. However, further metagenomic and metatranscriptomic analysis will be required to uncover both the rumen microbial genes and metabolic pathways associated with a low RME phenotype. Nonetheless, the abundance of a small cohort of rumen microbial genera has been identified as potential microbial biomarkers for methanogenesis, albeit the consistency of their relationship with RME will require further assessment across different diet types.

## Data Availability Statement

The datasets presented in this study can be found in online repositories. The names of the repository/repositories and accession number(s) can be found below: https://www.ncbi.nlm.nih.gov/, PRJNA797238.

## Ethics Statement

The animal study was reviewed and approved by Teagasc Animal Ethics Committee and licenced by Irish Health Products Regulatory Authority in accordance with European Union legislation (Directive 2010/63/EU), for the protection of animals used for scientific purposes.

## Author Contributions

SW, AK, DK, and PS conceived and designed the experiments and interpreted the results and wrote the manuscript. PS performed the experiments. PS and AK analysed the data. SW contributed reagents, materials, and analysis tools. All authors contributed to the article and approved the submitted version.

## Conflict of Interest

The authors declare that the research was conducted in the absence of any commercial or financial relationships that could be construed as a potential conflict of interest.

## Publisher’s Note

All claims expressed in this article are solely those of the authors and do not necessarily represent those of their affiliated organizations, or those of the publisher, the editors and the reviewers. Any product that may be evaluated in this article, or claim that may be made by its manufacturer, is not guaranteed or endorsed by the publisher.

## Footnotes

1
https://www.icbf.com/?page_id=12900

2
https://blast.ncbi.nlm.nih.gov/Blast.cgi?PAGE_TYPE=BlastSearch

## Supplementary Material

Supplementary File 1 Amplicon sequence variants (ASV) table of rumen bacteria and archaea genera identified in this study.
