# Supplementary material for: Differences in the Composition of the Rumen Microbiota of Finishing Beef Cattle Divergently Ranked for Residual Methane Emissions
Source: Front Microbiol. 2022 Apr 29;13:855565. doi: 10.3389/fmicb.2022.855565 (PMC9099143; doi:10.3389/fmicb.2022.855565)
Supplement: Supplementary file 1 [file Data_Sheet_1.DOCX]

***Supplementary Material***

| Supplementary Table 1. Details of the chemical composition of total mixed ration (TMR) and concentrates offered during feed efficiency and enteric emissions measurement periods (±SD) | | |
| --- | --- | --- |
|  | Concentrate | TMR |
| Chemical composition (% of DM unless stated) |  |  |
| Dry matter | 91.7 (0.8) | 50.1 (0.9) |
| Crude protein | 13.8 (0.4) | 12.2 (0.3) |
| Neutral detergent fibre | 21.8 (0.7) | 33.5 (1.1) |
| Acid detergent fibre | 10.8 (0.3) | 17.9 (0.6) |
| Either extract | 3.4 (0.6) | 2.3 (0.3) |
| Ash | 7.4 (0.2) | 7.3 (0.1) |
| Gross energy (MJ /kg DM) | 16.8 (0.3) | 16.7 (0.2) |

| **Supplementary Table 2** Characterization of enteric emissions and methane traits in finishing beef cattle ranked for residual methane emissions, sex and genotype. | | | | | | | | | | | | | | | | | | | | | | | | | | | | | | | | | | | | | | | | | | | | | | | | | | | | | | | |
| --- | --- | --- | --- | --- | --- | --- | --- | --- | --- | --- | --- | --- | --- | --- | --- | --- | --- | --- | --- | --- | --- | --- | --- | --- | --- | --- | --- | --- | --- | --- | --- | --- | --- | --- | --- | --- | --- | --- | --- | --- | --- | --- | --- | --- | --- | --- | --- | --- | --- | --- | --- | --- | --- | --- | --- |
|  |  |  | | | RME Ranking ^1^ | | | | | | |  | | | | | | | | | Sex | | | | | |  | | | | | | Genotype | | | |  | | | |  |  | | | |  | | |  | | | | | | |
| *Traits ^a^* | Mean ^2^ | | SD ^3^ | High | | | Medium | | | Low | | | | | SEM ^4^ | | | Steers | | | | | Heifers | | | | | | | SEM ^5^ | | Late | | | | Early | | SEM ^6^ | | | | | P-value | | | | | | | | P-value | | | P-value |  |
|  |  |  |  | *(n=84)* | | | *(n=114)* | | | *(n=84)* | | | | |  |  |  | *(n=128)* | | | | | *(n=154)* | | | | | | |  |  | *(n=219)* | | | | *(n=63)* | |  |  |  |  |  | *(RME Ranking)* | | | | | | | | *(Sex)* | | | *(Genotype)* |  |
| DME (g/day) | 229.2 | | 46.0 | 265.0^a^ | | | 224.0^b^ | | | 184.4^c^ | | | | | 8.8 | | | 232.0 | | | | | 217.0 | | | | | | | 12.1 | | 226.4 | | | | 222.5 | | 8.7 | | | | | <.0001 | | | | | | | | 0.38 | | | 0.30 |  |
| DCE (kg/day) | 8.4 | | 1.0 | 8.8^a^ | | | 8.3^b^ | | | 8.1^c^ | | | | | 0.2 | | | 8.6 | | | | | 8.2 | | | | | | | 0.3 | | 8.4 | | | | 8.4 | | 0.2 | | | | | <.0001 | | | | | | | | 0.39 | | | 0.92 |  |
| RME (g/day) | 0.00 | | 34.1 | 38.0^a^ | | | -0.1^b^ | | | -40.3^c^ | | | | | 1.8 | | | -0.7 | | | | | -0.9 | | | | | | | 1.8 | | 0.6 | | | | -2.2 | | 1.6 | | | | | <.0001 | | | | | | | | 0.94 | | | 0.25 |  |
| RME_CO2_ (g/day) | 0.00 | | 30.2 | 24.6^a^ | | | 0.7^b^ | | | -31.2^c^ | | | | | 2.4 | | | -1.2 | | | | | -2.7 | | | | | | | 2.3 | | 0.6 | | | | -4.6 | | 2.1 | | | | | <.0001 | | | | | | | | 0.65 | | | 0.11 |  |
| MY (g/ kg DMI ) | 22.10 | | 4.1 | 25.2^a^ | | | 21.6^b^ | | | 17.7^c^ | | | | | 0.7 | | | 21.9 | | | | | 21.1 | | | | | | | 1.0 | | 21.9^a^ | | | | 21.1^b^ | | 0.7 | | | | | <.0001 | | | | | | | | 0.59 | | | 0.01 |  |
| MADG (g/ kg ADG) | 171.7 | | 40.7 | 191.3^a^ | | | 167.1^b^ | | | 144.1^c^ | | | | | 6.6 | | | 166.8 | | | | | 168.1 | | | | | | | 8.5 | | 173.8^a^ | | | | 161.2^b^ | | 6.4 | | | | | <.0001 | | | | | | | | 0.91 | | | 0.02 |  |
| MI (g/ kg CW) | 0.70 | | 0.15 | 0.81^a^ | | | 0.67^b^ | | | 0.57^c^ | | | | | 0.03 | | | 0.68 | | | | | 0.69 | | | | | | | 0.05 | | 0.67^a^ | | | | 0.70^b^ | | 0.03 | | | | | <.0001 | | | | | | | | 0.83 | | | 0.01 |  |
|  | | | | | |  | | |  | | | | | | | | | | |  | |  | | | | | | |  | |  | | | | | | | |  | | | | | |  | | | | |  |  |  |  |  |  |
| ^a^ DME = daily methane production. DCE = daily carbon dioxide production. RME = residual methane emissions. RME_CO2_ = residual methane emissions calculated with carbon dioxide. MY = methane yield.. MI = methane intensity. | | | | | | | | | | |  | |  | | | |  | | | | |  |  |  |  |  |  |  |  |  |  |  |  |  |  |  |  |  |  |  |  |  |  |  |  |  |  |  |  |  |  |  |  |  |  |
| ^1^ High = RME was >0.5 SD above the mean; Medium = RME was±0.5 SD above and below the mean; Low = RME was >−0.5 SD below the mean. | | | | | | | | | | |  | |  | | | |  | | | | |  |  |  |  |  |  |  |  |  |  |  |  |  |  |  |  |  |  |  |  |  |  |  |  |  |  |  |  |  |  |  |  |  |  |
| ^2^ Overall trait mean. | | | | | | | |  | | | | | |  | |  | | |  | | | | |  | |  | | | | | | | | |  | | | | |  | | | | | | | |  | | | | |  |  |  |
| ^3^ Overall trait standard deviation. | | | | | | | |  | | | | | |  | |  | | |  | | | | | |  | | |  | | | | | |  | | | | | | | | | |  | | |  | | | | |  |  |  |  |
| ^4-6^ SEM = pooled standard error. | | | | | | | |  | | | | | |  | |  | | |  | | | | |  | |  | | | | | | | | |  | | | | |  | | | | | | | |  | | | | |  |  |  |
| ^a,b,c^ Least squares means within main effect and a row with different superscripts differ | | | | | | | |  | | | | | |  | |  | | |  | | | | |  | |  | | | | | | | | |  | | | | |  | | | | | | | |  | | | | |  |  |  |

| **Supplementary Table 3** Characterization of rumen fermentation profile in finishing beef cattle ranked for residual methane emissions, sex and genotype. | | | | | | | | | | | | | | | | | | | | | | | | | | | | | | | | | | | | | | | |  | |  |  |  | | | |
| --- | --- | --- | --- | --- | --- | --- | --- | --- | --- | --- | --- | --- | --- | --- | --- | --- | --- | --- | --- | --- | --- | --- | --- | --- | --- | --- | --- | --- | --- | --- | --- | --- | --- | --- | --- | --- | --- | --- | --- | --- | --- | --- | --- | --- | --- | --- | --- |
|  |  |  | | RME Ranking ^1^ | | | |  | | | | | Sex | | |  | | | | | | Genotype | |  | | | | | | |  | | | | | | | | | |  |  |  |  |  |  |  |
| *Rumen Fermentation ^a^* | Mean | SD | High | | Medium | Low | | | | SEM ^2^ | | Steers | | | Heifers | | | SEM ^3^ | | | Late | | Early | | SEM ^4^ | | P-value | | | P-value | | | | | | P-value | | | | |  |  |  |  |  |  |  |
|  |  |  | *(n=84)* | | *(n=114)* | *(n=84)* | | | |  |  | *(n=128)* | | | *(n=154)* | | |  |  |  | *(n=219)* | | *(n=63)* | |  |  | *(RME Ranking)* | | | *(Sex)* | | | | | | *(Genotype)* | | | | |  |  |  |  |  |  |  |
| pH | 6.8 | 0.3 | 6.8 | | 6.8 | 6.8 | | | | 0.1 | | 6.8 | | | 6.8 | | | 0.1 | | | 6.8 | | 6.8 | | 0.1 | | 0.48 | | | 0.76 | | | | | | 0.10 | | | | |  |  |  |  |  |  |  |
| Total SCFA (mM) | 124.2 | 34.4 | 134.5^a^ | | 120.9^b^ | 119.9^b^ | | | | 7.2 | | 119.8 | | | 130.4 | | | 8.9 | | | 123.4 | | 126.8 | | 6.9 | | 0.02 | | | 0.39 | | | | | | 0.54 | | | | |  |  |  |  |  |  |  |
| Acetate (%) | 74.3 | 6.7 | 73.6 | | 73.1 | 73.5 | | | | 1.5 | | 71.9 | | | 74.9 | | | 1.9 | | | 73.7 | | 73.1 | | 1.4 | | 0.85 | | | 0.26 | | | | | | 0.51 | | | | |  |  |  |  |  |  |  |
| Propionate (%) | 13.0 | 4.3 | 13.0^a^ | | 14.0^a^ | 14.5^b^ | | | | 1.2 | | 14.4 | | | 13.2 | | | 1.5 | | | 13.7 | | 13.9 | | 1.1 | | 0.04 | | | 0.59 | | | | | | 0.66 | | | | |  |  |  |  |  |  |  |
| Butyrate (%) | 7.8 | 2.6 | 8.0 | | 7.8 | 7.1 | | | | 0.7 | | 7.7 | | | 7.5 | | | 0.9 | | | 7.5 | | 7.7 | | 0.69 | | 0.10 | | | 0.89 | | | | | | 0.56 | | | | |  |  |  |  |  |  |  |
| A:P | 5.7 | 1.4 | 6.7^a^ | | 5.8^b^ | 5.7^b^ | | | | 0.6 | | 5.2 | | | 7 | | | 0.8 | | | 6.3 | | 5.9 | | 0.6 | | 0.03 | | | 0.11 | | | | | | 0.31 | | | | |  |  |  |  |  |  |  |
| Hydrogen Production (mM) | 663.7 | 157.2 | 688.6^a^ | | 622.9^b^ | 630.7^b^ | | | | 37.6 | | 626.5 | | | 668.3 | | | 48.0 | | | 654.8 | | 640.0 | | 36.5 | | 0.03 | | | 0.54 | | | | | | 0.56 | | | | |  |  |  |  |  |  |  |
|  |  |  |  | |  |  | | | |  | |  | | |  | | |  | | |  | |  | | | | |  |  | | | | | |  | | | |  | | | | | | | |  |
| ^a^ A:P = acetate to propionate ratio. | | | | | | | | | | | | | | | | | | | | | | | | | | | | | | | | |  | | | |  | | | | | | | |  |  |  |
| ^1^ High = RME was >0.5 SD above the mean; Medium = RME was±0.5 SD above and below the mean; Low = RME was >−0.5 SD below the mean. | | | | | | | | | | | | | | | | | | | | | | | | | | | | | | | | |  | | | |  | | | | | | | |  |  |  |
| ^2-4^ SEM = pooled standard error. | | | | | | |  | |  | |  | | |  | | |  | |  |  | | | | | |  | | | | | |  | |  | | | |  | | | | | | | |  |  |
| ^a,b,c^ Least squares means within main effect and a row with different superscripts differ. | | | | | | | | | | | | | |  | | |  | |  |  | | | | | |  | | | | | |  | |  | | | |  | | | | | | | |  |  |


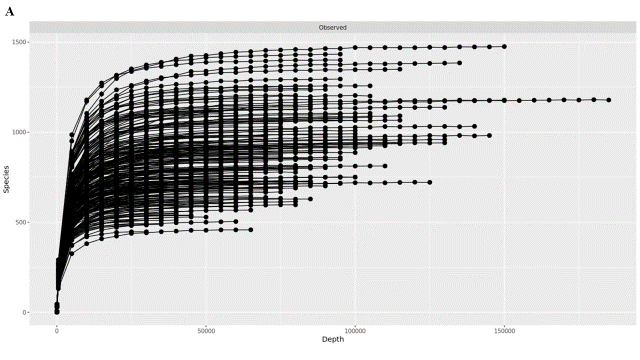


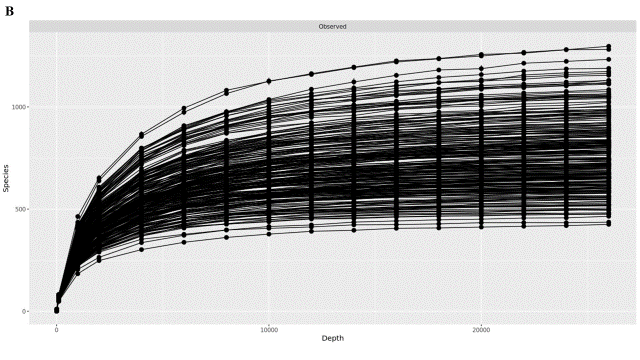
**Supplementary Figure 1** Rarefaction plot showing observed species from all samples before A) and after B) rarefication. Samples were rarefied to the lowest read depth of 26,366 reads.
